# Supplementary material for: Sensory Characteristics and Volatile Organic Compound Profile of Wild Edible Mushrooms from Patagonia, Argentina
Source: Foods. 2024 Oct 29;13(21):3447. doi: 10.3390/foods13213447 (PMC11545633; doi:10.3390/foods13213447)
Supplement: Supplementary file 1 [file foods-13-03447-s001.zip › foods-3252310-supplementary.pdf]

Supplementary material

Sensory characteristics and volatile organic compound profile of Wild Edible Mushrooms from Patagonia, Argentina

Barroetaveña, C.[1,2,3], González, C. G.[1,2], Tejedor-Calvo, E.[4,5], Toledo, C.[1], Pildain, M.B[1,2,3].

[1]Consejo Nacional de Investigaciones Científicas y Técnicas (CONICET)

[2]Área de Fitopatología y Microbiología Aplicada, Centro de Investigaciones y Extensión Forestal Andino Patagónico (CIEFAP). Ruta Nacional 259 km 16.24, Esquel, Chubut, Argentina.

[3]Universidad Nacional de la Patagonia San Juan Bosco (UNPSJB), Ruta 259 Km 16.4, 9200 Esquel, Chubut, Argentina.

[4]Department of Plant Science, Agrifood Research and Technology Centre of Aragon (CITA), Av. Montañana, 930, 50059 Zaragoza, Spain.

[5]Laboratory for Flavor Analysis and Enology (LAAE), Department of Analytical Chemistry, Universidad de Zaragoza, C/ Pedro Cerbuna 12 50009, Zaragoza, Spain

Corresponding author:

Gabriela C. González, ggonzalez@correociefap.org.ar

Eva Tejedor-Calvo, etejedor@unizar.es

Table S1. Sensory descriptor intensity evaluated for three sensory characteristics (Odor, Flavor and Texture) of nine wild edible mushroom species. Values are means of two replicates ± (SD). Mean values followed by different letters in each row indicate significant differences between the different species for the descriptors studied (p-value < 0.05).

	**Species**								
**Sensorial descriptors**	*G. vitellinum*	*P. dusenii*	*C. hariotii*	*C. magellanicus*	*F. antarctica*	*F. endoxantha*	*L. nuda*	*R. patagonica*	*G. gargal*
odor									
Almonds	0,40 (0,03)^{BC}	0,15 (0,12)^{C}	0,35 (0,03)^{BC}	0,20 (0,01)^{BC}	0,10 (0,03)^{C}	0,20 (0,21)^{BC}	0,70 (0,15)^{BC}	0,20 (0,23)^{BC}	3,95 (0,18)^{A}
Sweet	0,25 (0,12)^{A}	0,25 (0,01)^{A}	0,35 (0,01)^{A}	0,15 (0,02)^{A}	0,30 (0,21)^{A}	0,25 (0,06)^{A}	0,25 (0,04)^{A}	0,30 (0,03)^{A}	0,40 (0,14)^{A}
Spices	0,30 (0,02)^{B}	0,25 (0,03)^{B}	0,15 (0,03)^{B}	1,85 (0,12)^{A}	2,05 (0,03)^{A}	0,25 (0,12)^{B}	0,30 (0,23)^{B}	0,20 (0,04)^{B}	0,15 (0,21)^{B}
Musroom	3,90 (0,21)^{C}	5,35 (0,32)^{B}	2,00 (0,02)^{D}	5,80 (0,32)^{AB}	6,65 (0,12)^{A}	5,35 (0,21)^{B}	0,70 (0,01)^{E}	5,10 (0,02)^{B}	1,30 (0,01)^{DE}
Nuts	0,30 (0,01)^{C}	0,15 (0,02)^{C}	0,15 (0,03)^{C}	1,85 (0,02)^{B}	0,25 (0,17)^{C}	0,25 (0,02)^{C}	3,45 (0,03)^{A}	1,35 (0,06)^{B}	0,25 (0,03)^{C}
Wood	0,15 (0,20)^{B}	0,15 (0,21)^{B}	0,15 (0,14)^{B}	1,85 (0,03)^{A}	0,25 (0,21)^{B}	0,25 (0,03)^{B}	0,20 (0,21)^{B}	0,25 (0,04)^{B}	0,30 (0,21)^{B}
Earthy	0,45 (0,02)^{B}	0,25 (0,13)^{B}	0,15 (0,04)^{B}	1,90 (0,01)^{A}	0,40 (0,12)^{B}	0,30 (0,01)^{B}	1,05 (0,11)^{AB}	0,20 (0,15)^{B}	0,40 (0,02)^{B}
Flavor									

Almonds	0,20 (0,05)^{C}	0,15 (0,04)^{C}	0,20 (0,04)^{C}	1,05 (0,01)^{B}	0,30 (0,04)^{C}	0,20 (0,23)^{C}	0,45 (0,21)^{BC}	0,55 (0,02)^{BC}	3,35 (0,11)^{A}
Sweet	0,35 (0,01)^{DE}	0,35 (0,05)^{DE}	1,00 (0,03)^{CD}	0,20 (0,02)^{E}	7,00 (0,12)^{A}	4,60 (0,15)^{B}	1,65 (0,17)^{C}	6,25 (0,03)^{A}	0,25 (0,13)^{DE}
Spices	0,30 (0,03)^{A}	0,40 (0,01)^{A}	0,50 (0,02)^{A}	0,30 (0,03)^{A}	0,50 (0,01)^{A}	0,50 (0,03)^{A}	0,50 (0,03)^{A}	0,50 (0,04)^{A}	0,40 (0,21)^{A}
Nuts	0,25 (0,13)^{B}	0,35 (0,12)^{B}	0,40 (0,021)^{B}	1,60 (0,04)^{A}	0,50 (0,04)^{B}	0,50 (0,04)^{B}	0,45 (0,01)^{B}	0,55 (0,03)^{B}	0,50 (0,03)^{B}
Musroom	3,40 (0,04)^{A}	3,10 (0,15)^{AB}	1,50 (0,03)^{C}	0,40 (0,01)^{D}	0,15 (0,03)^{D}	0,40 (0,12)^{D}	2,45 (0,01)^{B}	0,30 (0,01)^{D}	0,20 (0,02)^{D}
Woody	0,40 (0,10)^{BC}	0,30 (0,11)^{BC}	0,80 (0,26)^{BC}	0,20 (0,12)^{C}	0,25 (0,01)^{BC}	0,35 (0,21)^{BC}	0,85 (0,03)^{B}	5,95 (0,23)^{A}	0,30 (0,01)^{BC}
Texture									
Soft	5,60 (0,04)^{B}	0,35 (0,02)^{C}	6,25 (0,12)^{A}	0,30 (0,03)^{C}	0,25 (0,05)^{C}	0,30 (0,21)^{C}	0,40 (0,13)^{C}	0,25 (0,12)^{C}	0,40 (0,21)^{C}
Fleshy	4,10 (0,16)^{C}	0,30 (0,05)^{F}	2,70 (0,25)^{D}	0,10 (0,12)^{F}	5,55 (0,31)^{B}	4,05 (0,05)^{C}	6,45 (0,19)^{A}	0,30 (0,04)^{F}	1,90 (0,02)^{E}
Cartilaginous	0,45 (0,05)^{C}	3,05 (0,31)^{B}	0,30 (0,12)^{C}	0,60 (0,04)^{C}	0,35 (0,02)^{C}	0,30 (0,04)^{C}	0,35 (0,02)^{C}	3,80 (0,03)^{A}	0,50 (0,21)^{C}
Leathery	0,35 (0,32)^{C}	0,30 (0,21)^{C}	0,50 (0,32)^{BC}	0,40 (0,03)^{BC}	0,35 (0,02)^{C}	1,00 (0,13)^{B}	0,25 (0,03)^{C}	0,30 (0,04)^{C}	3,35 (0,20)^{A}
Hard	0,20 (0,03)^{B}	0,30 (0,04)^{B}	0,30 (0,13)^{B}	0,40 (0,02)^{B}	0,25 (0,01)^{B}	0,25 (0,03)^{B}	4,45 (0,12)^{A}	0,30 (0,02)^{B}	0,40 (0,02)^{B}
Mucilaginous	0,30 (0,12)^{B}	0,30 (0,21)^{B}	0,30 (0,21)^{B}	2,30 (0,01)^{A}	0,50 (0,02)^{B}	0,30 (0,12)^{B}	0,30 (0,02)^{B}	0,35 (0,12)^{B}	0,25 (0,02)^{B}

Table S2. Sensory characteristics recorded on fresh specimens, refrigerated (Method I) and scalded and frozen (Method II) of wild mushroom edible species. nd: not detected

**Species**	**Preservation methods**	**Sensorial characteristics**			
		Odor	*Flavor*	*Texture*	*Color*
G. vitellinum	Fresh	Mushrooms	Mushrooms	Fleshy	Orange
	Method I	Mushrooms	Mushrooms	Fleshy	Orange
	Method II	Mushrooms	Mushrooms	Soft fleshy	Orange
P. dusenii	Fresh	Mushrooms	Mushrooms	Cartilaginous	pale ochraceous
	Method I	Mushrooms	Mushrooms	Cartilaginous	pale ochraceous
	Method II	Mushrooms	nd	Cartilaginous	pale ochraceous
C. hariotii	Fresh	Soft mushrooms	Sweet mushrooms	Soft fleshy	Yellow orange
	Method I	Mushrooms	Sweet mushrooms	Soft fleshy	Yellow orange
	Method II	Soft mushrooms	Sweet mushrooms	Soft fleshy	Yellow orange
C. magellanicus	Fresh	Hard mushrooms	Soft sweet	Mucilaginous	Bright lilac
	Method I	Hard mushrooms	Sweet	Mucilaginous	Light lilac
	Method II	Soft mushrooms	Sweet	Mucilaginous	Light lilac
F. antarctica	Fresh	Soft mushrooms	Sweet	Fleshy	Reddish
	Method I	Mushrooms	Sweet	Fleshy	Pale pink
	Method II	Soft mushrooms	Sweet	Soft fleshy	Pale pink
F. endoxantha	Fresh	Soft mushrooms	Sweet	Fleshy	Yellow chestnut
	Method I	Mushrooms	Sweet	Fleshy	Yellow chestnut
	Method II	Soft mushrooms	Sweet	Soft fleshy	Yellow chestnut
L. nuda	Fresh	Fruity	Soft mushrooms	Fleshy	Purplish blue
	Method I	Fruity	Mushrooms	Fleshy	Purplish blue
	Method II	Soft fruity	Soft mushrooms	Hard Fleshy	Purplish blue
R. patagonica	Fresh	Soft mushrooms	Wood	Velvety	Yellow
	Method I	Mushrooms	Soft wood	Velvety	Yellow
	Method II	Soft mushrooms	Sweet	Soft	Yellow
